# Subcortical Bone Cysts at the Medial Meniscus Posterior Root Are Associated with Longer Symptom Duration but Not with Healing Status or Meniscal Extrusion After Root Repair

**DOI:** 10.3390/medicina62050983

**Published:** 2026-05-18

**Authors:** Young-Mo Kim, Yong-Bum Joo, Young-Cheol Park

**Affiliations:** Department of Orthopedic Surgery, Chungnam National University Hospital, Chungnam National University College of Medicine, Munhwa-dong, Jung-gu, Daejeon 35015, Republic of Korea

**Keywords:** medial meniscus posterior root tear, bone cyst, meniscal extrusion, transtibial pullout repair, second-look arthroscopy

## Abstract

*Background and Objectives*: Subcortical bone cysts at the tibial attachment of the medial meniscus posterior root (MMPR) may reflect chronic degenerative changes; however, their clinical significance in medial meniscus posterior root tear (MMPRT) remains unclear. This study assessed whether bone cysts are associated with symptom duration and whether their presence influences healing status or medial meniscus extrusion (MME) after transtibial pullout repair (TPR). *Materials and Methods:* Seventy-four consecutive patients who underwent arthroscopic TPR for MMPRT between January 2022 and December 2024 were retrospectively reviewed. After applying exclusion criteria, 50 patients were included and divided into cyst-positive (*n* = 20) and cyst-negative (*n* = 30) groups based on preoperative MRI findings. Clinical outcomes, MME, and second-look arthroscopic healing status at 6 months postoperatively were compared between groups. Multivariable linear regression analysis was performed to identify independent predictors of postoperative MME. *Results*: The cyst-positive group had significantly longer symptom duration than the cyst-negative group (13.8 ± 3.0 vs. 8.8 ± 2.8 weeks, *p* < 0.001) and demonstrated higher grades of MMPR degeneration (*p* < 0.05). Complete healing was observed in 60.0% of patients in each group, and no failed healing cases were noted. Postoperative MME at 6 months was comparable between groups (3.8 ± 0.8 vs. 3.8 ± 1.0 mm). Multivariable regression analysis identified baseline MME as the strongest independent predictor of postoperative MME (β = 0.67, *p* < 0.001), whereas bone cyst presence was not independently associated with postoperative extrusion. *Conclusions*: Subcortical bone cysts at the MMPR attachment are associated with longer symptom duration and more advanced root degeneration. However, their presence was not significantly associated with healing status or postoperative MME after MMPRT repair. These findings suggest that bone cyst formation may be associated with chronic degenerative changes at the root attachment, but should not discourage surgeons from performing root repair.

## 1. Introduction

Medial meniscus posterior root tear (MMPRT) disrupts hoop tension and create a biomechanical condition comparable to total meniscectomy [1,2]. This leads to increased peak contact pressure in the medial compartment and accelerates degenerative changes in the knee [2]. Medial meniscus extrusion (MME), defined as medial displacement of the medial meniscus with respect to the central margin of the medial tibial plateau (MTP), is frequently observed in MMPRT and reflects loss of meniscal function [3,4]. Accordingly, arthroscopic transtibial pullout repair (TPR) has been widely performed to restore hoop tension and delay osteoarthritis progression [5,6,7].

Although repair improves clinical outcomes, progression of MME has still been reported [8,9,10,11]. Early surgical repair has therefore been emphasized, and intervention within 13 weeks from symptom onset has been suggested to minimize extrusion progression [12]. However, accurately determining the chronicity of MMPRT in clinical practice remains challenging.

Although some patients recall an acute painful popping event, others present with an insidious onset and minimal early symptoms [13,14]. Because symptom onset is often based on patient recall and mechanical symptoms are not consistently observed, the chronicity of MMPRT may be difficult to determine clinically [14]. It therefore remains unclear whether specific magnetic resonance imaging (MRI) findings at the root attachment reflect chronic stress and whether such chronicity influences postoperative healing status or MME.

Bone cysts are occasionally observed on MRI at the tibial attachment site of medial meniscus posterior root (MMPR) [15]. These subcortical cystic lesions, also described as insertional cysts, are thought to arise from focal bone resorption caused by chronic abnormal stress at ligament or meniscal attachment sites [16,17]. Accordingly, bone cyst formation at the root attachment may reflect chronic degenerative changes that develop prior to or independent of the root tear itself, rather than a direct consequence of untreated MMPRT. However, the clinical significance of these cysts and their relationship to symptom duration or postoperative outcomes remain unclear.

Therefore, the purpose of this study was to determine the clinical significance of bone cysts at the tibial attachment of the MMPR. Specifically, this study assessed whether the presence of subcortical bone cysts was associated with symptom duration and whether it influenced postoperative healing status or MME after TPR. It was hypothesized that bone cyst formation would be associated with longer symptom duration but would not adversely affect healing status or postoperative MME.

## 2. Materials and Methods

### 2.1. Study Population

From 1 January 2022 to 31 December 2024, a total of 74 consecutive patients underwent arthroscopic TPR for MMPRT at our institution. This study was approved by the Chungnam National University Hospital Institutional Review Board (CNUH 2026-03-004; approval date: 23 January 2026). Informed consent was not required due to the retrospective nature of the study. All patients were diagnosed with MMPRT on preoperative MRI. MMPRT were considered present if at least two of the following three MRI signs were identified: (1) absence of an identifiable meniscal root or a high signal replacing the normal dark meniscal signal on sagittal imaging (ghost sign); (2) a radial linear defect at the meniscal root on axial imaging; and (3) a vertical linear defect at the meniscal root on coronal imaging [4]. Photographs obtained during arthroscopic surgery were reviewed to confirm the diagnosis of MMPRT and to evaluate other intra-articular lesions. All medical records were retrospectively reviewed to obtain demographic and clinical characteristics of the enrolled patients. The inclusion criteria were as follows: (1) chronic symptomatic root tear without diffuse chondral lesions; (2) International Cartilage Repair Society (ICRS) grade ≤ 2, or focal ICRS grade 3 lesions amenable to microfracture; (3) Kellgren–Lawrence (KL) grade ≤ 3; (4) desire to return to the previous activity level; (5) willingness to use crutches for at least 6 weeks postoperatively; (6) willingness to follow the rehabilitation protocol and avoid deep knee flexion; and (7) meniscal tissue suitable for fixation without complex tear patterns or severe degenerative changes.

**Figure 1:**
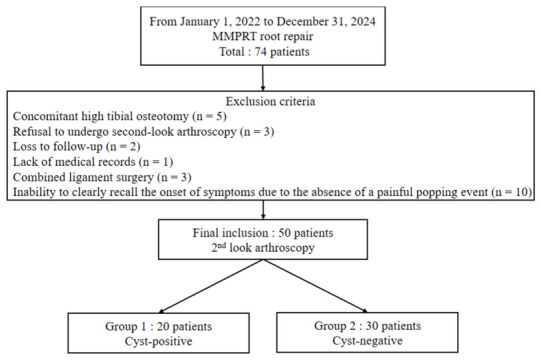
Flowchart of included patients. MMPRT: medial meniscus posterior root tear.

Of the 74 patients who underwent arthroscopic TPR for MMPRT during the study period, 24 were excluded for the following reasons: concomitant high tibial osteotomy (*n* = 5), refusal to undergo second-look arthroscopy (*n* = 3), loss to follow-up (*n* = 2), lack of medical records (*n* = 1), combined ligament surgery (*n* = 3), and inability to clearly recall the onset of symptoms due to the absence of a painful popping event (*n* = 10). Finally, 50 patients were included in the study. Symptom duration was defined as the interval from the onset of symptoms, identified by a patient-recalled painful popping event, to the time of preoperative MRI acquisition. All included patients underwent second-look arthroscopic evaluation at 6 months postoperatively (Figure 1).

### 2.2. Surgical Technique

**Figure 2:**
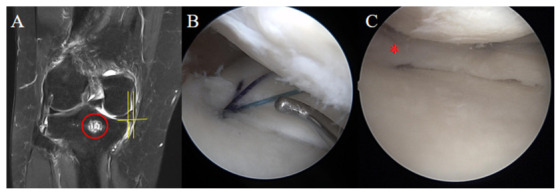
Representative Case of MMPRT Repair and Second-Look Arthroscopy. (**A**) Preoperative coronal T2-weighted MRI of a 55-year-old woman with a painful popping event, showing medial meniscus posterior root tear with medial meniscus extrusion (3.52 mm). A red circle indicates the subcortical bone cyst at the tibial attachment site. Yellow lines demonstrate the method used to measure medial meniscus extrusion. (**B**) Arthroscopic view during transtibial pullout repair. According to the tibial tunnel position, the torn root was repaired using a simple vertical suture configuration. (**C**) Second-look arthroscopy performed 6 months postoperatively. The red asterisk (*) indicates the repaired medial meniscus posterior root, which demonstrated partial healing with maintained continuity but increased laxity on probing. MMPRT: medial meniscus posterior root tear.

All surgical procedures were performed by a single senior surgeon using a TPR technique. The choice between a remodified Mason–Allen suture (rMMA) configuration and a simple vertical suture was determined according to the tibial tunnel position [18]. (Figure 2B) Of the 20 patients in the cyst-positive group, 17 underwent repair using the rMMA configuration and 3 underwent simple vertical suture. Of the 30 patients in the cyst-negative group, 25 underwent repair using the rMMA configuration and 5 underwent simple vertical suture.

**Figure 3:**
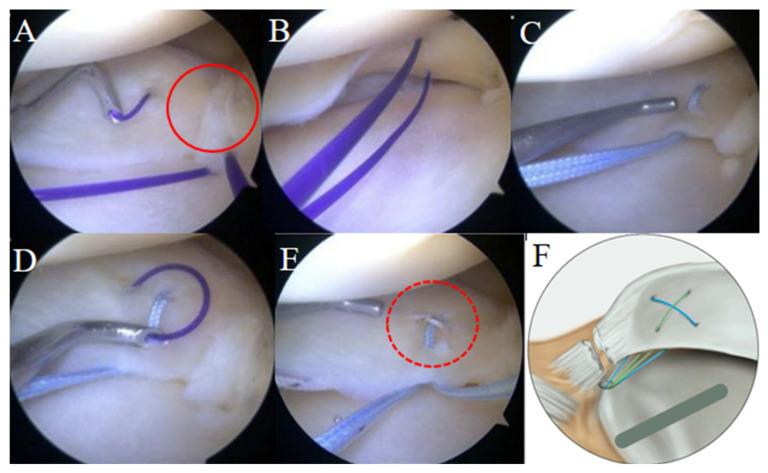
Remodified Mason–Allen Suture Configuration for MMPRT Repair. (**A**) A Caspari curved suture hook loaded with a PDS suture is passed through the medial meniscus posterior root from the superior to the inferior surface. A red circle indicates the torn end of the medial meniscus posterior root. The first suture passage is maintained while preparing for a second pass through the meniscus. (**B**) Two strands of PDS sutures are visible on the undersurface of the medial meniscus posterior root after sequential passage using the Caspari suture hook. (**C**) The PDS sutures are replaced with FiberWire sutures using the shuttle relay technique to create a horizontal loop suture configuration. (**D**) Using the same technique, a vertical loop suture is placed across the horizontal loop to complete the locking configuration. (**E**) The remodified Mason–Allen suture configuration is completed using two FiberWire sutures. The red dashed circle highlights the cross-shaped configuration formed by the intersection of the vertical and horizontal loops. (**F**) Schematic illustration of the remodified Mason–Allen stitch configuration used for medial meniscus posterior root repair. MMPRT: medial meniscus posterior root tear; PDS: Polydioxanone suture.

After diagnostic arthroscopy confirmed the MMPRT, the torn meniscal root and adjacent posterior capsular tissue were debrided using a rasp and motorized shaver to obtain fresh meniscal tissue. A Caspari curved suture hook loaded with a No. 2–0 polydioxanone synthetic (PDS (Ethicon, Somerville, NJ, USA) suture was introduced through the anterolateral portal (Figure 3A). With the knee flexed approximately 10–30° and slight valgus force applied, the suture hook was passed from the superior to the inferior surface of the medial meniscus posterior horn (MMPH), approximately 5–7 mm medial to the torn edge. While maintaining the first PDS strand in place, the hook was passed again anterior to the first suture point, allowing two strands of PDS to be positioned beneath the undersurface of the MMPH (Figure 3B). Using the shuttle-relay technique, the PDS sutures were replaced with No. 2 FiberWire sutures (Arthrex, Naples, FL, USA), and a horizontal loop suture was created (Figure 3C). Subsequently, a vertical loop suture was made between approximately 5–7 mm and 7–9 mm medial to the torn edge, crossing over the horizontal loop to complete a cross-shaped loop configuration (Figure 3D,E).

A 2.4 mm guide pin was introduced through an anterior cruciate ligament tibial guide and positioned at the anatomic root attachment site. A tibial tunnel measuring 5–6 mm in diameter was created along the guide pin using a cannulated drill bit. All FiberWire sutures were passed through the tibial tunnel, and the torn meniscus was anatomically reduced by pulling the sutures. Final fixation was performed with the knee flexed at approximately 30°, and a 7 × 23 mm biocryl interference screw was inserted into the tibial tunnel to secure the sutures under appropriate tension.

### 2.3. Postoperative Rehabilitation

After surgery, the knee was immobilized in full extension with a locked hinged knee brace in all patients. Immobilization was maintained for 6 weeks postoperatively, during which non-weight bearing was strictly enforced. Range of motion (ROM) exercises were initiated from postoperative day 1 but limited to 45° of flexion during the first 6 weeks. At 7 to 8 weeks postoperatively, ROM was gradually increased to 90° of flexion, and toe-touch weight-bearing ambulation using crutches was allowed. At 9 to 10 weeks, flexion was permitted up to 110°, and patients progressed to 50% partial weight-bearing with crutch-assisted gait. Between 11 and 12 weeks postoperatively, full flexion was allowed, and full weight-bearing ambulation was initiated. Return to sports activities was permitted at 6 months postoperatively. Squatting and deep knee flexion were strictly prohibited until 6 months after surgery. Throughout the rehabilitation period, quadriceps strengthening exercises, straight leg raising, and ankle pump exercises were encouraged as tolerated. The same standardized rehabilitation protocol was applied to all patients.

### 2.4. Clinical Outcomes

The Lysholm score, International Knee Documentation Committee (IKDC) subjective knee form, and visual analog scale (VAS) for pain were assessed as clinical outcome measures [19,20,21]. Preoperative clinical scores were obtained on the day before surgery, and final clinical scores were recorded at the last follow-up visit. The clinical scores in each group were compared with their respective final results. Additionally, the final results within each group were compared. Clinical evaluations were assessed by one of the authors who was blinded to the surgical data.

### 2.5. Plain Radiographs Evaluation

Knee radiographs were obtained at the first outpatient clinic visit. Mechanical axis alignment was evaluated using full-length standing radiographs. The mechanical axis was defined as the line connecting the center of the femoral head to the center of the ankle joint. Varus alignment was recorded as positive (+) values, and valgus alignment was recorded as negative (−) values [4]. Degenerative changes in the medial compartment were assessed using the KL grading system on a standing 45° flexion posteroanterior weight-bearing view of the knee (Rosenberg view) [22]. In this grading system, grade 0 represents a normal joint; grade 1, doubtful joint space narrowing and possible osteophytic lipping; grade 2, definite osteophytes with possible joint space narrowing; grade 3, definite joint space narrowing with multiple osteophytes and sclerosis; and grade 4, marked joint space narrowing with severe sclerosis and definite deformity of the bone ends [4,23]. Joint space width (JSW) was measured at the center of the medial and lateral compartments on the Rosenberg view. Medial joint space width (JSW) was additionally measured using the midpoint technique. The midpoint technique measures the medial JSW with a line passing through the location defined by the midpoint of the medial tibial eminence and the medial margin of the MTP, drawn parallel to the long axis of the tibia [24,25]. Posterior tibial slope (PTS) was measured on lateral radiographs using the tibial proximal anatomic axis (TPAA). The TPAA was defined as a line connecting the central points of the tibial cortex at 5 cm and 15 cm below the tibial tuberosity. The line connecting the highest anterior and posterior points of the MTP was regarded as the tibial slope line. The angle between the tangent to the MTP and the line perpendicular to the TPAA was defined as the PTS [26].

### 2.6. Magnetic Resonance Imaging Evaluation

All MRI examinations were performed using 1.5-T or 3.0-T superconducting scanners with patients in the supine position without weight-bearing. Not all examinations were performed on the same scanner, as some patients had undergone MRI at outside institutions prior to referral. Standard knee MRI protocols were used at all facilities, and measurements were performed in a consistent manner to minimize inter-institutional variability. MRI was assessed for MMPR-related findings, including subcortical cystic lesions, the extent of MME, and the severity of degeneration (defined as central intrameniscal signal not communicating with the superior or inferior margin).

A bone cyst at the MMPR attachment site was specifically defined as a well-defined intraosseous lesion located just below the posterior root insertion of the medial meniscus and anterior medial to the posterior cruciate ligament. This lesion demonstrated high signal intensity on T2-weighted coronal and sagittal images and low signal intensity on T1-weighted images [15]. MME was measured at the point of maximal extrusion at the level of the medial collateral ligament. MME was defined as the distance between a vertical line drawn at the outer margin of the MTP (excluding osteophytes) and a vertical line drawn at the outer margin of the medial meniscus. An extrusion greater than 3 mm was considered pathologic [3,4]. MME was evaluated at three time points: preoperatively, postoperative 3 days, and at 6 months postoperatively. The change in extrusion was calculated as follows: Δ1 (preoperative MME − postoperative day 3 MME) and Δ2 (6-month MME − postoperative day 3 MME). The severity of medial meniscal degeneration was assessed using the MRI-based grading system described by Kim et al., which classifies degeneration based on the T2 signal intensity of the meniscus: grade 0 (pure black); grade 1 (predominantly black with mild white signal); grade 2 (white signal <50%); grade 3 (white signal >50%); and grade 4 (predominantly white with mild black signal) [27].

All radiologic measurements were performed using a picture archiving and communication system workstation (Maroview, version 5.4.10.52; Marotech, Seoul, Republic of Korea). Measurements were performed three times at 4-week intervals by observers blinded to clinical information, and mean values were used for analysis.

### 2.7. Meniscus Root Healing Status Through Second-Look Arthroscopy

With patient consent, a second arthroscopy was performed at 6 months postoperatively. The same surgeon carried out both the root repair and the second-look arthroscopy. The healing status of the repaired root was classified as complete, partial, or failed. The healing states were defined with slight modifications of the classifications described by Kim et al. and Seo et al.: complete healing = meniscal continuity with no cleft and sufficient tension without lifting on probing; partial healing = good meniscal continuity, with some loss of tension and apparent increased lifting on probing; and failed healing = no continuity, no tension on probing, and no evidence of healing [28,29]. No failed healing cases were observed in the study cohort.

During arthroscopy, the condition of the cartilage was examined according to the ICRS classification, in which grade I indicates that the cartilage exhibits superficial, blunt gaps, as well as superficial cracks; grade II, the cartilage damage depth is less than 50% of the cartilage depth; grade III, the cartilage damage depth is greater than 50% of the cartilage depth but does not reach the subchondral bone; and grade IV, there is a total cartilage tear with exposure of the subchondral bone [30].

### 2.8. Statistical Analysis

All statistical analyses were performed using SPSS software (version 23.0; IBM Corp., Armonk, NY, USA). The level of statistical significance was set at *p* < 0.05. Continuous variables were expressed as mean ± standard deviation and were compared using the independent *t* test or Mann–Whitney U test, as appropriate. Categorical variables were compared using the chi-square test or Fisher’s exact test. To determine independent predictors of 6-month MME, multivariable linear regression analysis was performed. Variables included in the model were age, BMI, preoperative varus alignment, baseline MME, symptom duration, and the presence of a bone cyst. The regression coefficient (β) and 95% confidence interval (CI) were calculated. The overall goodness of fit of the model was assessed using the coefficient of determination (R^2^).

## 3. Results

A total of 50 patients who underwent TPR for MMPRT were included in this study. Among them, 20 patients were classified into the bone cyst–positive group and 30 into the bone cyst–negative group. There were no significant differences between the two groups with respect to age (59.5 ± 5.1 vs. 57.4 ± 10.2 years), BMI (25.3 ± 2.8 vs. 27.4 ± 4.0 kg/m^2^), preoperative varus alignment (2.2 ± 1.5° vs. 2.5 ± 1.7°), or preoperative MME (3.5 ± 0.5 mm vs. 3.3 ± 1.1 mm). However, the duration from symptom onset to MRI was significantly longer in the cyst-positive group (13.8 ± 3.0 weeks) compared with the cyst-negative group (8.8 ± 2.8 weeks, *p* < 0.001). Regarding categorical variables, no significant differences were observed between groups in sex distribution, laterality, or KL grade distribution.

The distribution of MMPR degeneration grades differed significantly between the two groups (*p* < 0.05). The cyst-positive group demonstrated a higher proportion of advanced degeneration (grades 3 and 4), whereas the cyst-negative group showed a higher proportion of mild degeneration (Table 1).

In terms of healing status at follow-up, complete healing was observed in 12 patients (60.0%) in the cyst-positive group and 18 patients (60.0%) in the cyst-negative group. The remaining patients demonstrated partial healing, and no failed healing was observed in either group. There was no statistically significant difference in healing status between the two groups.

Preoperative MME did not differ significantly between groups. Postoperative day 3 MME values were also comparable (1.20 ± 0.8 mm vs. 1.3 ± 1.1 mm). At 6 months postoperatively, MME was nearly identical between the cyst-positive and cyst-negative groups (3.8 ± 0.8 mm vs. 3.8 ± 1.0 mm). Similarly, the degree of MME change from preoperative to postoperative day 3 measurements (Δ1) and from postoperative day 3 to 6 months (Δ2) did not significantly differ between groups (Table 2).

Statistically significant improvements were observed in all clinical outcomes, including the Lysholm score, IKDC subjective score, and VAS pain score, in both groups (all *p* < 0.05) (Table 3).

Multivariable linear regression analysis was performed to identify independent predictors of 6-month MME. After adjusting for age, BMI, preoperative varus alignment, baseline MME, and duration from symptom onset to MRI, the presence of a bone cyst was not independently associated with 6-month postoperative MME. Baseline MME was the strongest independent predictor of 6-month MME (β = 0.67, *p* < 0.001). Increasing age was also significantly associated with greater MME at 6 months (β = 0.03, *p* = 0.017). Other variables, including BMI, varus alignment, and duration from symptom onset to MRI, were not significant predictors. The overall model was statistically significant (R^2^ = 0.575, *p* < 0.001) (Table 4).

## 4. Discussion

The most important finding of this study was that subcortical bone cysts at the MMPR attachment were associated with longer symptom duration and higher grades of MMPR degeneration. However, the presence of bone cysts was not significantly associated with healing status or postoperative MME after TPR in this study. These findings suggest that bone cyst formation may reflect chronic mechanical stress at the root attachment rather than a factor that determines postoperative structural outcomes. To our knowledge, few studies have evaluated the relationship between subcortical bone cysts at the MMPR attachment and postoperative healing status and MME after TPR. The present study provides additional clinical insight into the potential role of bone cysts in surgical decision-making for MMPRT.

Bone cysts at the tibial attachment site are thought to result from focal bone resorption caused by chronic abnormal stress at ligament or meniscal insertion sites [16]. Son et al. reported that among 52 subcortical cysts identified at the posterior subspinous region of the tibia, 48 were located at the medial meniscus posterior horn root insertion and all of them (100%) were associated with adjacent meniscal pathology [16]. From a biomechanical perspective, MMPRT disrupts hoop tension and increases contact pressure in the medial compartment [1,2]. This chronic mechanical overload at the tibial root attachment may contribute to subchondral bone changes and cyst formation, which may be associated with longer symptom duration and more advanced meniscal degeneration [16].

In the present study, bone cysts were observed in 20 of 50 patients (40%), and the cyst-positive group had a significantly longer symptom duration than the cyst-negative group (13.8 ± 3.0 vs. 8.8 ± 2.8 weeks). Omae et al. also reported that the duration from symptom onset to MRI was longer in the cyst-positive group than in the cyst-negative group (12.9 ± 13.1 vs. 8.3 ± 10.9 weeks). [15] Kim et al. investigated intraosseous cysts in patients with MMPR degeneration, partial tears, and complete tears, and reported that cystic lesions were most frequently observed in the degeneration group [4]. Because their study included degenerative root lesions as well as tears, the onset of symptoms may have been less clearly defined. In contrast, the present study included arthroscopically confirmed MMPRT, and many patients could recall a painful popping event, allowing a more consistent estimation of symptom duration.

Patients in the cyst-positive group also demonstrated more advanced MMPR degeneration. The cyst-positive group showed a higher distribution of degeneration grades compared with the cyst-negative group (3/5/8/4 vs. 15/8/5/2, *p* < 0.05). These findings suggest that cyst formation may be associated with a more advanced degenerative environment at the root attachment. Previous studies have also indicated that degenerative changes in the MMPR are closely related to chronic overload of the medial compartment. In particular, longer disease duration has been associated with greater MME and higher grades of subchondral insufficiency fracture of the knee (SIFK) [31]. Because all patients in this cohort experienced a distinct painful popping event suggestive of MMPRT onset, MRI examinations were obtained after the occurrence of the root tear. Previous studies have reported that a single episode of painful popping is highly predictive of MMPRT and often represents the moment of root failure during daily activities [13]. Therefore, the higher degeneration grades observed in the cyst-positive group may reflect more advanced degenerative changes present around the time of the tear. In this context, cyst formation around the root attachment may reflect chronic degenerative changes that develop over time, but the temporal relationship with the occurrence of MMPRT remains uncertain.

Bone cysts were associated with longer symptom duration and more advanced MMPR degeneration; however, these findings did not appear to prevent structural healing after repair. Healing status was comparable between the cyst-positive and cyst-negative groups. Notably, no failed healing cases were observed in either group. These findings suggest that restoration of root continuity may be achievable even in the presence of chronic degenerative changes.

However, restoration of root continuity does not necessarily indicate restoration of normal meniscal tension. Chung et al. reported intact root healing in 97% of patients after root repair using modified Mason–Allen stitches, but only 56% of those demonstrated non-lax tissue on probing during second-look arthroscopy [23]. Seo et al. reported that no case achieved complete healing using simple sutures, whereas Kim et al. observed lax healing in all cases during second-look arthroscopy [28,29]. In addition, Moon et al. reported that 34 of 63 patients (54%) showed complete healing after arthroscopic TPR using a modified reverse Mason–Allen stitch technique [12].

These findings indicate that restoration of root continuity can frequently be achieved even in degenerative root tears. However, due to the intrinsic elasticity of the meniscus and degenerative tissue quality, repetitive loading may gradually elongate the repaired root, resulting in insufficient restoration of tension. In the present study, the rMMA technique was used; nevertheless, the overall pattern of healing reported in previous studies using different suture configurations—such as simple vertical stitches, modified Mason–Allen stitches, and modified reverse Mason–Allen stitches—appears to be broadly similar. This suggests that the biological environment of the degenerative meniscus, rather than the specific suture configuration itself, may play a more important role in determining the quality of healing after root repair.

MME has been considered one of the key structural changes associated with MMPRT and reflects the biomechanical consequence of loss of hoop tension [11,32,33]. Geissbuhler et al. reported that MME is closely related to degenerative structural changes in the meniscus and may even precede the development of MMPRT in some cases [11]. However, the relationship between symptom duration and MME remains unclear. Kim et al. analyzed patients with MMPRT and reported that symptom duration was not significantly correlated with MME (*p* = 0.722) [34]. This finding suggests that the duration of symptoms may not reliably reflect the progression of MME, particularly because symptom onset is often defined by a painful popping event and may not correspond to the actual timing of structural degeneration [12,13]. Preoperative MME itself may play a more important role in determining postoperative MME. Kamatsuki et al. reported that the preoperative MME was significantly greater in patients whose MME increased after repair than in those whose MME decreased (5.0 ± 1.1 mm vs. 3.8 ± 1.1 mm, *p* = 0.045) [35].

These findings suggest that the extent of MME observed after MMPRT repair may depend largely on the preexisting structural condition of the meniscus rather than the duration of symptoms.

From a clinical perspective, although bone cyst formation was associated with longer symptom duration and more advanced root degeneration, it did not appear to adversely affect healing status or postoperative MME after repair. This suggests that the presence of bone cysts should not necessarily discourage surgeons from performing root repair. Previous studies have demonstrated that degenerative MMPRT can still benefit from surgical repair compared with nonoperative or meniscectomy treatment [36]. In a systematic review and meta-analysis by Krivicich et al., radiographic osteoarthritis progression occurred in 22% (18/82) of patients who underwent root repair compared with 66% (41/62) of those who underwent meniscectomy (OR, 0.17; *p* = 0.029). Furthermore, conversion to total knee arthroplasty (TKA) occurred in 9.8% (8/82) of patients after repair compared with 36% (22/61) after meniscectomy (OR, 0.15; *p* < 0.001) [36]. These findings indicate that preservation of the meniscal root through repair may delay degenerative progression and reduce the risk of subsequent TKA. Therefore, the presence of bone cysts may reflect chronic mechanical stress around the root attachment but does not appear to preclude restoration of root continuity after repair.

This study has several limitations. First, this was a single-center retrospective study, and selection bias cannot be completely excluded. Second, the sample size was relatively small, which may limit the generalizability of the findings and precluded subgroup analyses by degeneration grade to evaluate its potential effect on healing quality. Third, although second-look arthroscopy was performed at 6 months postoperatively in all included patients, longer follow-up is required to evaluate long-term structural and clinical outcomes, including radiographic osteoarthritis progression and conversion to TKA. Fourth, symptom duration was determined based on patient recall of a painful popping event, which may introduce recall bias and may not precisely reflect the actual onset of structural degeneration. Fifth, two suture configurations were used depending on tibial tunnel position. Although no formal comparison between techniques was performed, the distribution was similar between groups (cyst-positive: 17 rMMA configurations, three simple vertical suture configurations; cyst-negative: 25 rMMA configurations, five simple vertical suture configurations), which limits the likelihood of differential confounding. Sixth, no adjustment for multiple comparisons was applied, which may increase the risk of statistical error and should be considered when interpreting the results. In addition, although healing status was assessed arthroscopically at second-look arthroscopy, the functional consequences of partial healing were not separately analyzed in this study, and therefore remain unclear. Finally, MRI examinations were not performed on a standardized scanner, as some patients had undergone imaging at outside institutions using either 1.5-T or 3.0-T machines, which may have introduced variability in image quality and potentially affected diagnostic accuracy.

Future prospective studies with larger sample sizes, standardized MRI protocols, and longer follow-up periods are warranted to further clarify the clinical significance of subcortical bone cysts in MMPRT and their relationship to postoperative outcomes.

## 5. Conclusions

Subcortical bone cysts at the MMPR attachment were associated with longer symptom duration and higher grades of MMPR degeneration. However, the presence of bone cysts was not significantly associated with healing status or postoperative MME after MMPRT repair. These findings suggest that bone cyst formation may be associated with chronic degenerative changes at the root attachment. Importantly, the presence of bone cysts should not necessarily discourage surgeons from performing root repair, as restoration of root continuity may be achievable even in cyst-positive cases.

## Figures and Tables

**Table 1:** Comparison of variables between the two groups classified according to the presence of bone cyst.

Variables	Group1 (Cyst Positive, *n* = 20)	Group2 (Cyst Negative, *n* = 30)	*p*-Value
Demographic data			
Age, years	59.5 ± 5.1	57.4 ± 10.2	n.s. ^a^
Sex: male/female	3/17	5/25	n.s. ^b^
Affected side, left/right	9/11	13/17	n.s. ^b^
Body mass index	25.3 ± 2.8	27.4 ± 4.0	n.s. ^a^
Duration from symptom onset to MRI, weeks	13.8 ± 3.0	8.8 ± 2.8	<0.001 ^a^
Radiologic data			
Mechanical axis (varus), °	2.2 ± 1.5	2.5 ± 1.7	n.s. ^b^
Posterior tibial slope, °	10.6 ± 1.7	10.4 ± 2.1	n.s. ^a^
Medial joint space, mm	4.3 ± 1.0	4.3 ± 1.1	n.s. ^a^
Medial meniscus extrusion, mm	3.5 ± 0.5	3.3 ± 1.1	n.s. ^a^
Kellgren-Lawrence grade, 1/2/3/4	6/11/3/0	9/16/5/0	n.s. ^b^
MMPR degeneration grade, 1/2/3/4	3/5/8/4	15/8/5/2	<0.05 ^b^

Data are presented as mean ± standard deviation or number of patients. BMI, body mass index; MRI, magnetic resonance imaging; MME, medial meniscus extrusion; MMPR, medial meniscus posterior root. n.s., not significant. ^a^ Compared using the independent *t* test or Mann–Whitney U test, as appropriate based on normality assessment. ^b^ Compared using Fisher’s exact test.

**Table 2:** Comparison of the postoperative healing status and radiologic data between group 1 and 2.

Variables	Group1 (Cyst Positive, *n* = 20)	Group2 (Cyst Negative, *n* = 30)	*p*-Value
Healing status			
Complete/Partial/Failed	12/8/0	18/12/0	n.s. ^b^
Radiologic data			
Postoperative 3 days medial meniscus extrusion, mm	1.2 ± 0.8	1.3 ± 1.2	n.s. ^a^
Postoperative 6 months medial meniscus extrusion, mm	3.8 ± 0.8	3.8 ± 1.0	n.s. ^a^
∆Medial meniscus extrusion 1, mm	2.3 ± 1.0	2.0 ± 1.1	n.s. ^a^
∆Medial meniscus extrusion 2, mm	2.6 ± 1.2	2.5 ± 1.1	n.s. ^a^

Data are presented as mean ± standard deviation or number of patients. n.s., not significant. ΔMME 1 = preoperative MME − postoperative day 3 MME; ΔMME 2 = 6-month postoperative MME − postoperative day 3 MME. ^a^ Compared using the independent *t* test or Mann–Whitney U test, as appropriate based on normality assessment. ^b^ Compared using Fisher’s exact test.

**Table 3:** Preoperative and postoperative outcomes according to presence of bone cyst.

Clinical Scores	Preoperative	Final Follow-Up	*p*-Value
Group1(cyst positive)			
Lysholm score	52.3 ± 14.5	75.7 ± 13.5	<0.05 ^a^
IKDC score	39.1 ± 15.2	63.7 ± 15.1	<0.05 ^a^
VAS score	6.3 ± 1.5	2.6 ± 0.9	<0.05 ^a^
Group2(cyst negative)			
Lysholm score	52.4 ± 15.8	73.5 ± 14.1	<0.05 ^a^
IKDC score	41.2 ± 17.5	61.6 ± 13.4	<0.05 ^a^
VAS score	6.4 ± 1.7	2.8 ± 0.5	<0.05 ^a^

Data are presented as mean ± standard deviation. IKDC, International Knee Documentation Committee; VAS, visual analog scale. ^a^ Compared using the paired *t*-test (preoperative vs. final follow-up within each group).

**Table 4:** Multivariable linear regression analysis for postoperative medial meniscus extrusion.

Variables	β Coefficient	95% CI	*p*-Value
Bone cyst	−0.13	−0.65 to 0.40	n.s.
Age	0.03	0.00 to 0.06	0.017
Body mass index	0.04	−0.02 to 0.10	n.s.
Mechanical axis (varus), °	−0.004	−0.13 to 0.12	n.s.
Preoperative medial meniscus extrusion, mm	0.67	0.44 to 0.90	<0.001
Duration from symptom onset to MRI, weeks	−0.002	−0.07 to 0.06	n.s.

β, unstandardized regression coefficient. CI, confidence interval; BMI, body mass index; MRI, magnetic resonance imaging; MME, medial meniscus extrusion. n.s., not significant. Overall model: R^2^ = 0.575, *p* < 0.001.

## Data Availability

The data that support the findings of this study are available from the corresponding author upon reasonable request.

## Abbreviations

The following abbreviations are used in this manuscript:

MMPRT	Medial meniscus posterior root tear
MME	Medial meniscus extrusion
MTP	Medial tibial plateau
TPR	Transtibial pullout repair
rMMA	Remodified Mason-Allen
MRI	Magnetic resonance imaging
MMPR	Medial meniscus posterior root
MMPH	Medial meniscus posterior horn
ICRS	International Cartilage Repair Society
KL	Kellgren–Lawrence
PDS	Polydioxanone synthetic
ROM	Range of motion
IKDC	International Knee Documentation Committee
VAS	Visual analog scale
JSW	Joint space width
PTS	Posterior tibial slope
TPAA	Tibial proximal anatomic axis
CI	Confidence interval
SIFK	Subchondral insufficiency fracture of the knee
TKA	Total knee arthroplasty
